# Efficacy and safety of Venetoclax-based regimens in relapsed or refractory multiple myeloma: a systematic review and meta-analysis of prospective clinical trials

**DOI:** 10.1080/07853890.2023.2186480

**Published:** 2023-03-13

**Authors:** Wei He, Fang He, Huixian Hu

**Affiliations:** Department of Hematology, Affiliated Jinhua Hospital, Zhejiang University School of Medicine, Jinhua, Zhejiang, People’s Republic of China

**Keywords:** BCL-2 inhibitor, t(4;14), BCL-2 expression, regimen, adverse event

## Abstract

**Background:**

Multiple myeloma (MM) is an incurable malignancy. Venetoclax (VEN) shows a meaningful effect in MM patients who are relapsed or refractory (RR) to previous standard therapies.

**Objective:**

This study aimed to assess the efficacy and safety of VEN-based treatments in RR MM patients.

**Materials and methods:**

Comprehensive studies were searched in PubMed, Embase, Web of Science and Cochrane library. Efficacy was assessed by overall response rate (ORR), strict complete response rate (sCR), complete response rate (CR), very good partial response rate (VGPR) and partial response rate (PR).

**Results:**

Seven studies containing 482 subjests were included. The pooled ORR, ≥ CR (sCR + CR), VGPR and PR were 68% (51%–85%), 24% (13%–35%), 25% (17%–34%) and 17% (11%–24%) respectively. Multi-drug treatments were superior to VEN ± dexamethasone (Dex) treatments in ORR (82% vs 42%, *p* = .003) and ≥ CR (36% vs 7%, *p* < 0.00001). Subgroup analysis indicated patients achieve higher ORR who harboring t(11;14) translocation or containing high BCL-2 expression.

**Conclusions:**

VEN-containing regimens could be suggested as effective and safe treatments to RR MM patients with t(11;14) or high BCL-2 levels.

## Introduction

Multiple myeloma (MM) is an incurable plasma cell malignancy and characterized by bone destruction, anemia, renal failure and hypercalcemia [1,2]. Newly diagnosed MM patients are usually treated with bortezomib (a first-generation proteasome inhibitor (PI)), lenalidomide (a second-generation immunomodulatory drug (IMiD)) and dexamethasone (Dex), which is the current standard of care [3,4]. A portion of eligible patients could undergo frontline autologous stem cell transplantation (ASCT) to acquire prolonged remission [5,6]. Daratumumab, a monoclonal antibody targeting CD38, shows superior response rates and progression-free survival (PFS) compared with traditional therapies [7,8]. The prognosis of MM has been improved dramatically however there are still several incurable cases [9]. In the relapsed/refractory (RR) setting, MM becomes increasingly aggressive and overall survival (OS) might less than 1 year [10]. Innovative therapeutic agents with a novel mechanism of action are needed urgently. Members of the BCL-2 family are important anti-apoptosis regulators, including BCL-2, BCL-XL and MCL-2, and regarded as attractive targets for therapy [11,12].

Venetoclax (VEN), the first oral BCL-2 inhibitor, shows a promising prospect in the treatment of RR MM. BCL-2 has been found over-expressed in a subset of MM, particularly in those harboring t(11;14) [13,14]. With an occurrence rate of 15%–20%, t(11;14) is one of the commonest chromosome translocation in MM [15]. *In vitro* studies show VEN could induce the disruption of BCL-2/BAX, BCL-2/BIK, or BCL-2/Puma complexes, and then kill MM cell lines [16,17]. In addition, VEN can augment the efficacy of immune checkpoint inhibitors by increasing PD-1 positive T effector memory cells in mouse syngeneic tumor models [18]. Besides, VEN also proved meaningful clinical activity in RR MM [19].

In this study, we aimed to evaluate the safety and efficacy of VEN-based therapies in RR MM. Furthermore, we used subgroup analysis to identify whether therapeutic strategies that combine VEN with other agents are associated with improved efficacy (VEN + Dex + other agents vs VEN ± Dex).

## Methods

### Literature search

We conducted a search in PubMed, Embase, Web of Science and Cochrane library on January 4, 2022. The search strategy is as follows: ‘Venetoclax’ and ‘multiple myeloma’. There were no date, language or article type restrictions.

### Inclusion and exclusion criteria

**Figure 1.**
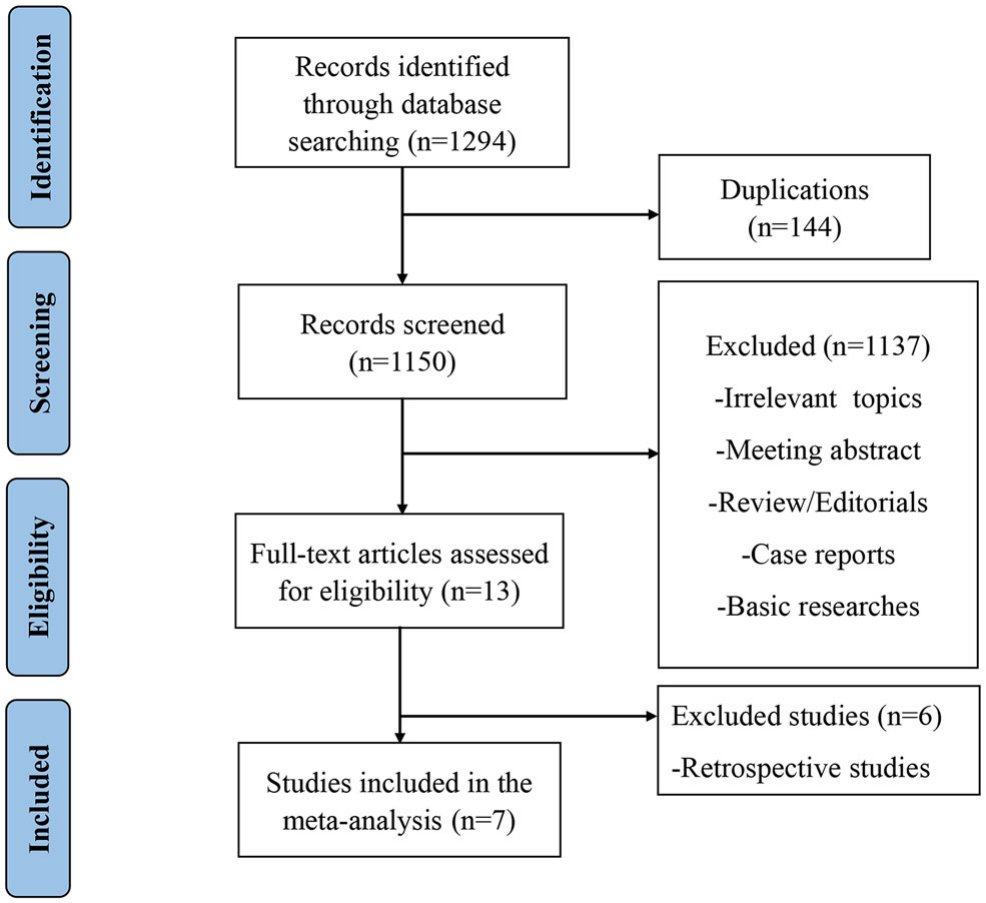
Flow diagram for the identification of the studies.

Studies were included if they met the following criteria: (1) clinical trials of VEN contained regimens for patients with RR MM; (2) studies had reported certain treatment outcomes and adverse effects (AEs). We excluded irrelevant topics, meeting abstract, reviews, editorials, case reports, basic researches and retrospective studies. In addition, different articles focused on the same trial at varied time points were regarded as the same one, and only the most recent study was included. The strategies of literature selection were described in Figure 1. Two authors searched and selected studies independently, and discussed with any conflicts.

### Data extraction

Two investigators reviewed and extracted the following data separately: author, year, the phase of the study, ClinicalTrials.gov number, number of patients, median age, sex, the median number of prior lines of treatments, previous regimens exposed (IMiD, PI, anti-CD38 monoclonal antibody and ASCT), cytogenetics (t(11;14), high BCL-2 expression), intervention (VEN dose and therapies with other drugs), efficacy outcomes (overall response rate (ORR), strict complete response rate (sCR), complete response rate (CR), very good partial response (VGPR), partial response rate (PR)) and AEs. Discrepancies were dealt with consultation with the supervisor.

### Statistical analysis

Revman 5.3 was applied to analyze therapeutic efficacy and safety. We used the I^2^ index to evaluate the heterogeneity of the studies (<25%, low heterogeneity, 25–50%, moderate heterogeneity, and >50%, significant heterogeneity). If the heterogeneity was significant, a random effects model was used while a fixed one was applied when the heterogeneity was moderate or low. Subgroup analysis was also conducted to assess treatment effects according to the varied clinical profiles.

### Study qualitative assessment

Considering the majority clinical trials in our analysis were single-armed, the Methodological Index for Non-randomized Studies (MINORS) was adopted to evaluate the quality of included studies. There are 12 items of MINORS and 8 of them were specified for non-comparative studies [20]. The items were scored 0 (not reported), 1(reported butinadequate), or 2 (reported and adequate). The randomized study was assessed using the Cochrane Collaboration’s tool to evaluate the quality of the included study [21].

## Results

### Literature search and characteristics

1294 potentially relevant publications were retrieved preliminarily. 144 duplicates were removed. Other 1137 records were excluded for review, case reports, retrospective studies, meetings, basic researches etc. Seven studies containing 482 subjects met the inclusion criteria [19,22–27]. Figure 1 exhibits the PRISMA flow diagram of study selection.

The characteristics of the included studies were summarized in Table 1. All studies were single-arm clinical trials except one. The dosage of VEN ranged from 50 to 1200 mg daily. 117 patients in 2 studies were treated with VEN ± Dex while 365 patients in 5 studies were taken three or more drugs regimen (VEN + Dex + other targets like PI, IMiD and Daratumumab). Patients included in our research at least had one line therapy. More than half received stem cell transplantation. The mean age of patient ranged from 63 to 67.5 years and the proportion of t (11;14) was 14–100%. The quality of included RCT study was assessed for bias risk by the Cochrane Collaboration tool (Supplementary Figure S1). Other studies were evaluated by MINORS scores (Table 1). All studies stated a clearly aim, included consecutive patients, prospectively collected data, had appropriate endpoints and follow-up period. The loss of follow-up was less than 5%. No study reported blind evaluation of endpoints. 6 studies (75%) did not prospectively calculate the size of the study. In general, the quality of included studies was adequate.

**Table 1.** Characteristics of the included studies.

Study	Design	Treatment	No. of patients	Age range	Prior lines of therapy	Prior ASCT (%)	t (11;14) (%)	ORR	mPFS(m)	MINORs scores
Kaufman [25], phase1	phase 1	VenDex	20	46–77	3(1–8)	85%	100%	60%	12.4	12
Kaufman [25], phase2	phase 2	VenDex	31	48–80	5(2–12)	58%	100%	48%	10.8	12
Kumar [19]	phase 1	Ven/VenDex	66	31–79	5(1–15)	76%	45%	21%	2.6	14
Bahlis [22], part1	phase 1	DaraVenDex	24	51–76	2.5(1–8)	63%	100%	95.80%	NR	12
Bahlis [22], part2	phase 1	DaraBortVenDex	24	41–80	1(1–3)	50%	25%	91.70%	NR	12
Kumar [26]	phase 3	BortVenDex	194	59–73	2(1–3)	60%	10%	82%	22.4	RCT
Costa [23]	phase 2	CFZVenDex	49	37–79	1(1–3)	51%	27%	80%	22.8	12
Gasparetto [24]	phase 2	PomVenDex	8	60–77	1.5(1–5)	75%	38%	63%	10.5	12
Moreau [27]	phase 1b	BortVenDex	66	38–79	3(1–13)	59%	14%	67%	9.5	14

Abbreviations: Ven: venetoclax; Bort: bortezomib; Dex: dexamethasone; Len: lenalidomide; Pom: pomalidomide; CFZ: carfilzomib; IMiD: immunomodulatory, PI: proteasome inhibitor, ORR: overall response rate; PFS: progression-free survival; NA: not available; NR: not reached.

### Efficacy

**Figure 2.**
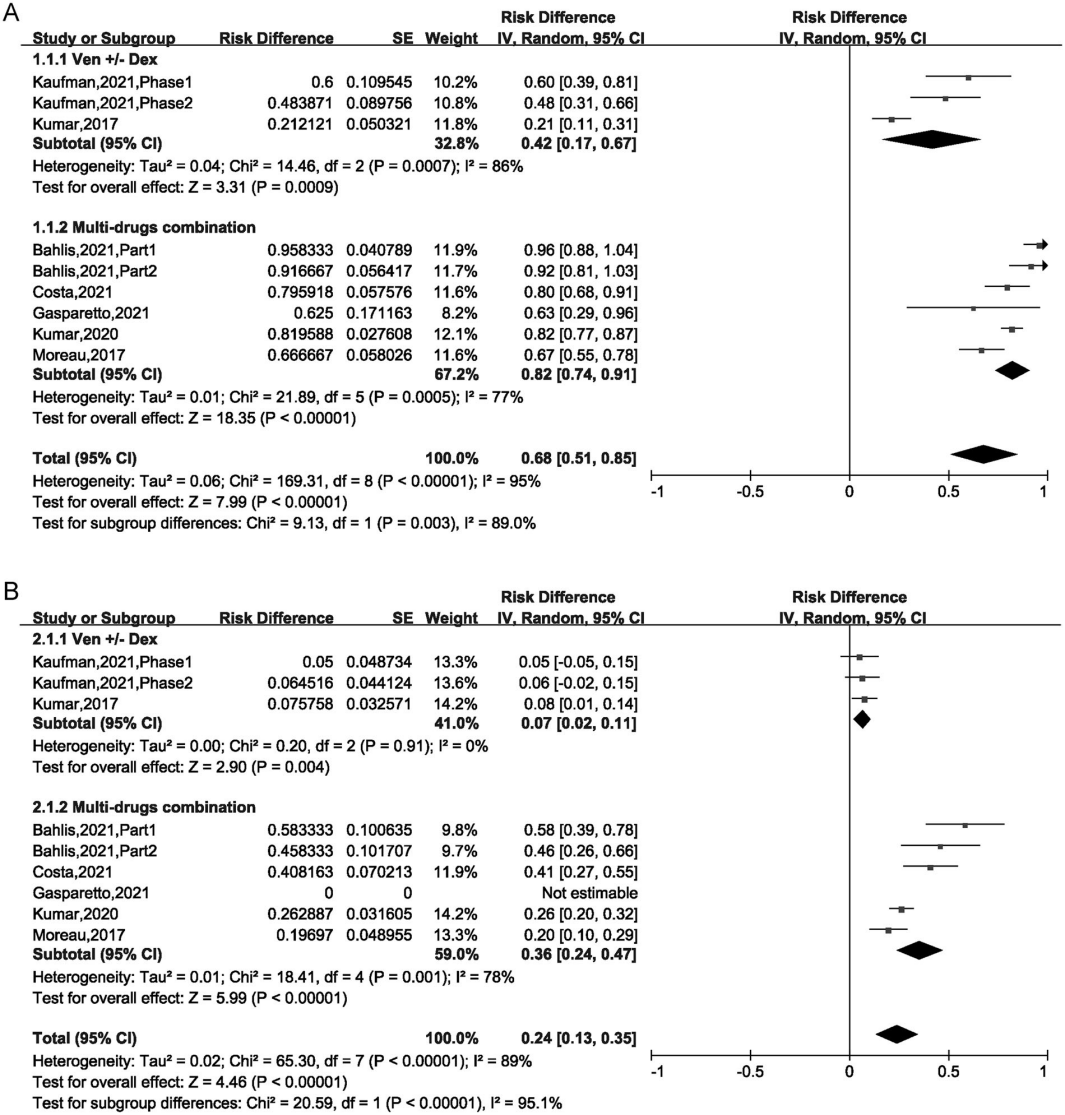
The forest plot of pooled (A) ORR and (B) ≥ CR.

The ORR fluctuated between 21% and 95.8% among the included 9 trials, and the pooled ORR was 68% (95% CI: 51%–85%), indicated that almost 68% patients achieved PR or better to VEN-based treatment. For the VEN ± Dex regimen, the ORR was 42% (95% CI: 17%–67%), for the VEN + Dex + other targets regimen, the ORR was 82% (95% CI: 74%–91%). This result suggested that the regimen of the VEN + Dex + other targets was superior compared with the regimen of the VEN ± Dex (82% vs 42%, *p* = .003) (Figure 2).

**Figure 3.**
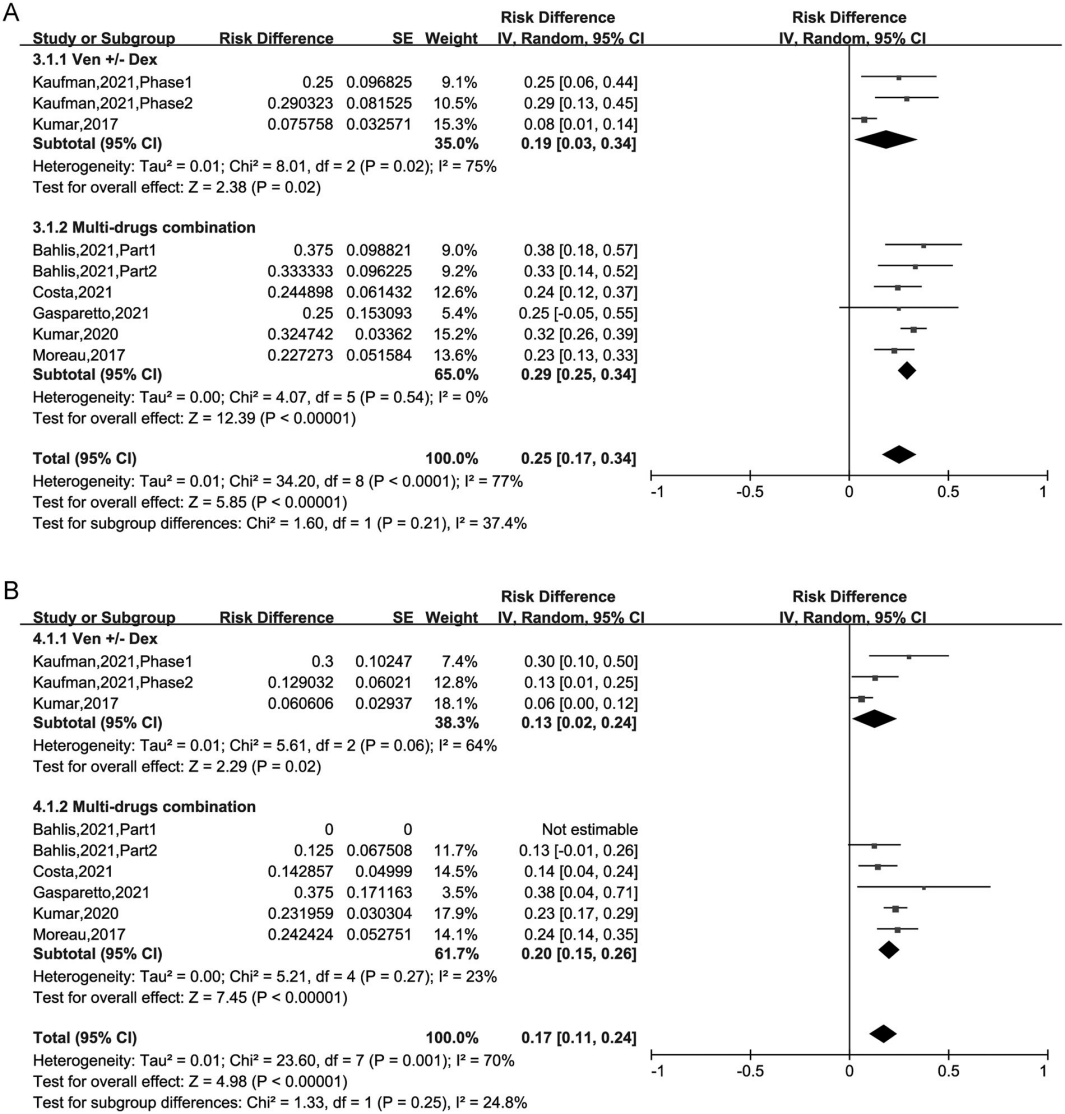
The forest plot of pooled (A) VGPR and (B) ≥ PR.

The pooled ≥ CR was 24% (95% CI: 13%–35%). Subgroup analysis suggested that CR was 7% (95% CI: 2%–11%) for patients treated with VEN ± Dex, 36% (95% CI: 24%–47%) for those treated with VEN + Dex + other targets. This result revealed that the CR of patients using VEN + Dex + other targets treatment was higher than in those using VEN ± Dex treatment (36% vs 7%, *p* < .00001) (Figure 2). The pooled VGPR was 25% (95% CI: 17%–34%) and PR was 17% (95% CI: 11%–24%) respectively, as showed in Figure 3. Sub-analysis has no significant difference between the two regimens in VGPR and PR group (Figure 3).

Subgroup analysis was subsequently conducted to evaluate different characteristics of the trials treated with VEN + Dex + other targets therapy (Table 2). ORR was 92% (95% CI: 87%–98%) in patients harboring t(11;14) and 78% (95% CI: 68%–89%) in those without this translocation. Besides, ORRs of 88% and 71% were observed in patients with high and low BCL-2 expression. The results showed that patients with t(11;14) or high BCL-2 levels could achieve higher ORR (92% vs 78%, *p* = .02; 88% vs 71%, *p* = .03). There is no significant difference of ORR occurred in the median age (≤65 years vs >65 years), previous therapy lines (≤2 lines vs >2 lines) and the proportion of ASCT patients (<60% vs ≥60%).

**Table 2.** Subgroup analysis of ORR.

Subgroup	No. of trials	ORR	95% CI	*p* for differences
Median age (y)				
≤65	3	0.85	0.68,1.02	.67
>65	3	0.81	0.76,0.86	
Previous therapy lines				
≤2	4	0.83	0.77,0.89	.92
>2	2	0.82	0.53,1.10	
Previous ASCT rate (%)				
<60%	3	0.79	0.65,0.94	.53
≥60%	3	0.86	0.72,0.99	
t(11,14)				
With	6	0.92	0.87,0.98	.02
Without	5	0.78	0.68,0.89	
BCL-2 expression				
High	3	0.88	0.82,0.94	.03
Low	3	0.71	0.55,0.86	

### Safety

**Figure 4.**
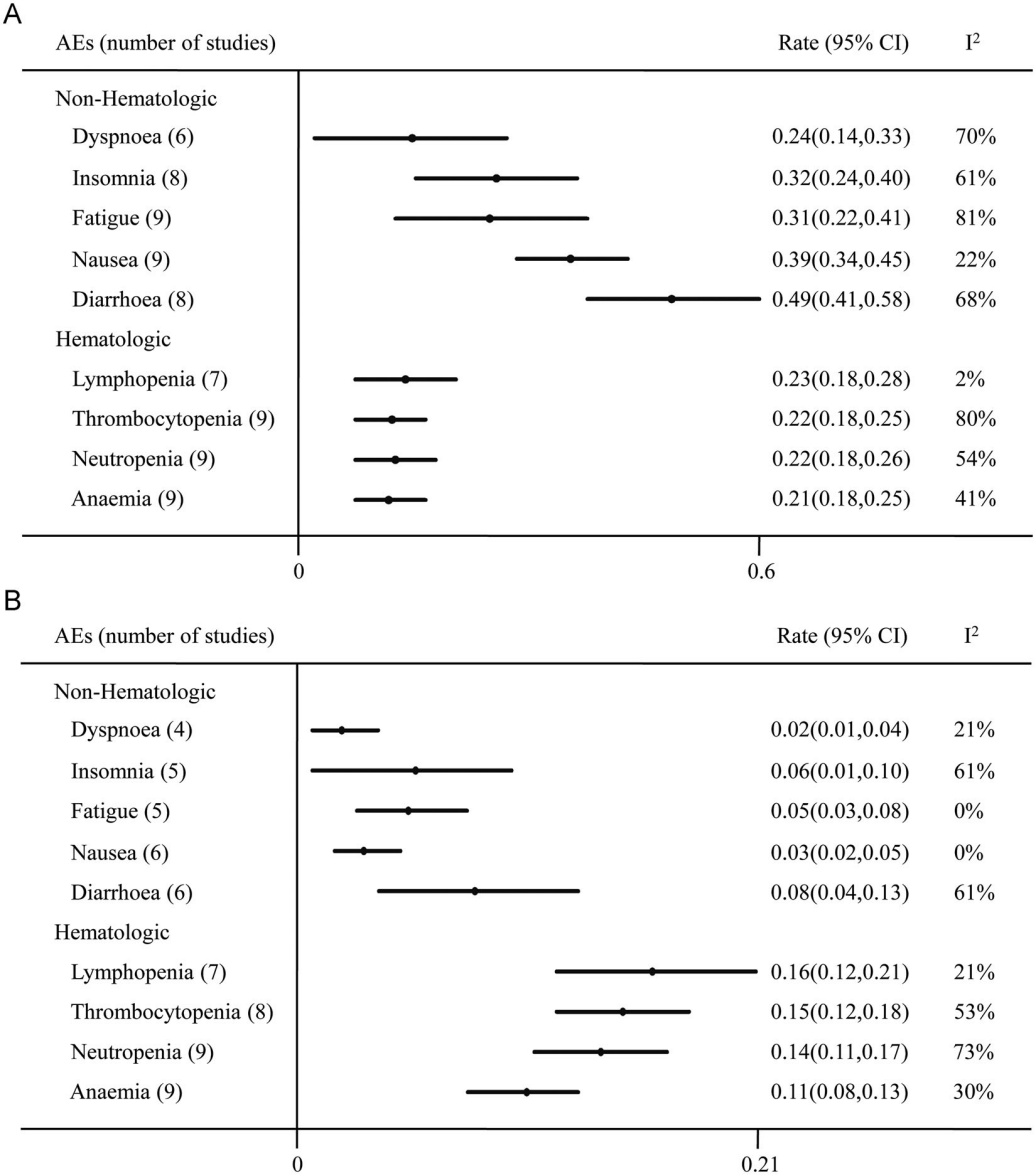
Meta analysis of AEs. (A) The most common AEs. (B) Grade 3 or higher AEs.

Digestive tract reaction and myelosuppression were the frequently reported AEs for the use of VEN. The most common hematological AEs included lymphopenia (23%), thrombocytopenia (22%), neutropenia (22%) and anaemia (21%) (Figure 4). The leading non hematological AEs were diarrhea (49%), nausea (39%), insomnia (32%), fatigue (31%), dyspnoea (24%). Mean incidences of grade 3 or higher hematologic AEs were lymphopenia (16%), thrombocytopenia (15%), neutropenia (14%), anaemia (11%) and nonhematologic AEs were diarrhea (8%), insomnia (6%), fatigue (5%), nausea (3%), dyspnoea (2%).

### Treatment dose and schedule

2 trials designed a range of VEN doses to determine the maximum tolerated dose (MTD). In one of the trial, Moreau et al. utilized VEN at 50–1200 mg daily and the MTD was not reached [27]. ≥VGPR response rates increased with the dosages of VEN through 800 mg while Grade 3/4 neutropenia increased simultaneously. In another trial, daily VEN given at doses up to 1200 mg also showed acceptable safety [19]. In the BELLINI trial, 194 patients received 800 mg VEN orally daily of each 21-d cycles. The dose of VEN was reduced in 32% patients for AEs [26]. 7 trials used VEN at 800 mg or 400 mg VEN once daily. The dosage of VEN should be reduced for the concomitant use of CYP3A inhibitors.

## Discussion

The prognosis of MM has been dramatically developed during the past decades [28]. However, clinical heterogeneity led to variable outcomes of patients and some individuals relapsed and remained incurable [29,30]. Consequently, it is urgent to develop novel treatments with differential mechanisms.

We included 7 studies and a total of 482 MM patients in this systematic review and meta-analysis. The results indicated that treatment with VEN increased the response rate and prolonged survival of RR MM patients who were resistant to PIs, IMiDs, anti-CD38 antibody and ASCT previously. The pooled analysis showed 68% (95% CI: 51%–85%) patients could achieve ORR. Furthermore, we conducted a separate analysis of different regimens. The ORR was higher in the multi-combination of the VEN group than in VEN ± Dex group (82% vs 42%, *p* = .003). We also tried to identify the best regimen however the sample size was limited. More prospective trials should be performed to deal with this question. Kumar et al. used VEN at doses up to 1200 mg and found the dosage was generally safe and well tolerated in the majority [19]. Some patients had VEN dose reduction for AEs. Most patients of the studies administered VEN 400 mg or 800 mg daily. The dose of VEN should be reduced with the concomitant use of moderate or strong CYP3A inhibitors. Pharmacokinetic experiments showed peal concentrations of VEN are achieved at 2–8 h after administration and did not be affected by co-treatment of daratumumab or bortezomib [22].

The phase1 Study of VEN monotherapy has shown high response rates in patients with RR MM containing t(11;14), which is considered an intermediate risk marker [19]. This study enrolled 66 patients who had received a median of five lines of therapy and 21% (14/66) of them had attained ORR. Most responses (12/14 (86%)) were reported in patients containing t(11;14). The ORR in t(11;14) patients was 40% (12/30). Kaufman et al. also showed VEN improved clinical outcomes of RR MM patients harboring t(11;14) who had failed treatment with an anti-CD38 monoclonal antibody [25]. Patients with t(11;14) were enrolled in this trial and 87% of them were refractory to daratumumab therapy previously. After a median follow-up of 9.2 months, the ORR was 48%. Besides, the phase 3 BELLINI trial implied that patients with high BCL-2 expression and/or t(11;14) translocation had better response rate and longer progression-free survival in the VEN group than those in the placebo group [26]. The rate of VGPR or better in patients with high BCL-2 expression was 71% (47/66) with VEN and 28% (9/32) with placebo. Compared with those 17 patients expressing low BCL-2, Costa et al. observed higher ORR (86% vs 65%) and MRD negativity (18% vs 12%) rate in 22 BCL-2^high^ patients [23]. The median time to disease progression (11.6 vs 5.7 months) and duration of overall response (10.2 vs 7 months) were also longer in patients with high BCL-2 expression [27].

These findings might provide the new possibility to determine personalized regimens according to certain marker. Because MM is a complex disease with high variability, VEN is more efficacious against MM cells that are more dependent on BCL-2 for proliferation [14,31]. The dependency of BCL-2 is different between patients and influenced by the existence of genetic abnormalities (i.e. t(11;14)) and expression of the BCL-2 protein family. Subgroup analysis in our meta also shows better response rates in groups with BCL-2 high levels or t(11;14) translocation.

The most common hematological AEs in all grades were lymphopenia, thrombocytopenia, neutropenia and anaemia. Furthermore, the most common non hematological AEs were diarrhea, nausea, insomnia, fatigue and dyspnoea. These AEs could be managed by supportive care and dose modification. The BELLINI trial observed that a substantial proportion of patients suffered early death for infection in the VEN group, compared with the placebo group [26]. Subgroup analysis showed the increased mortality occurred in patients without t(11;14) and with low BCL-2 expression. The potential reason might be the absent response to therapy, concomitant disease deterioration and treatment-related immunosuppression. All these factors induce patients susceptible to life-threatening infection. It is suggested MM individuals with VEN-based treatments should have the implementation of antibiotic prophylaxis. Oral and intravenous hydration was employed to mitigate the risk of tumor lysis syndrome (TLS) so that there were rare reports of TLS. Only 3 cases were observed who experienced TLS [25].

There are several limitations in our review and meta-analysis which should be taken into consideration. First, the majority of studies included were single-arm clinical trials and only one was a phase 3 trial. Second, some included studies had limited population size. Furthermore, clinical heterogeneity existed among studies such as different regimens, varied VEN dosage, number of prior lines of therapy, percentage of patients with t(11;14) or high BCL-2 expression or high-risk cytogenetic abnormalities. We observed that the regimens of the VEN + Dex + other targets were more effective than VEN ± Dex. However, the patients on the VEN + Dex had received more prior lines of therapy who might be refractory to current therapies. Fourth, potential publication bias could influence our result.

## Conclusion

VEN exhibited notable efficacy and manageable safety in patients with RR MM. It is a promising option for its unique effect of BCL-2 inhibition and may offer a novel biologic-driven approach in MM. The multi-drug regimens of VEN showed an improved effect than VEN + Dex regimen. Patients with t(11;14) and high BCL-2 expression were susceptible to the drug. Further studies are warranted to design the optimal regimen.

## Supplementary Material

Supplemental Material

## Data Availability

No data are available in this study.
