# Supplementary figures and images for: Efficacy and safety of Venetoclax-based regimens in relapsed or refractory multiple myeloma: a systematic review and meta-analysis of prospective clinical trials
Source: Ann Med. 2023 Mar 13;55(1):1029–36. doi: 10.1080/07853890.2023.2186480 (PMC10795640; doi:10.1080/07853890.2023.2186480)

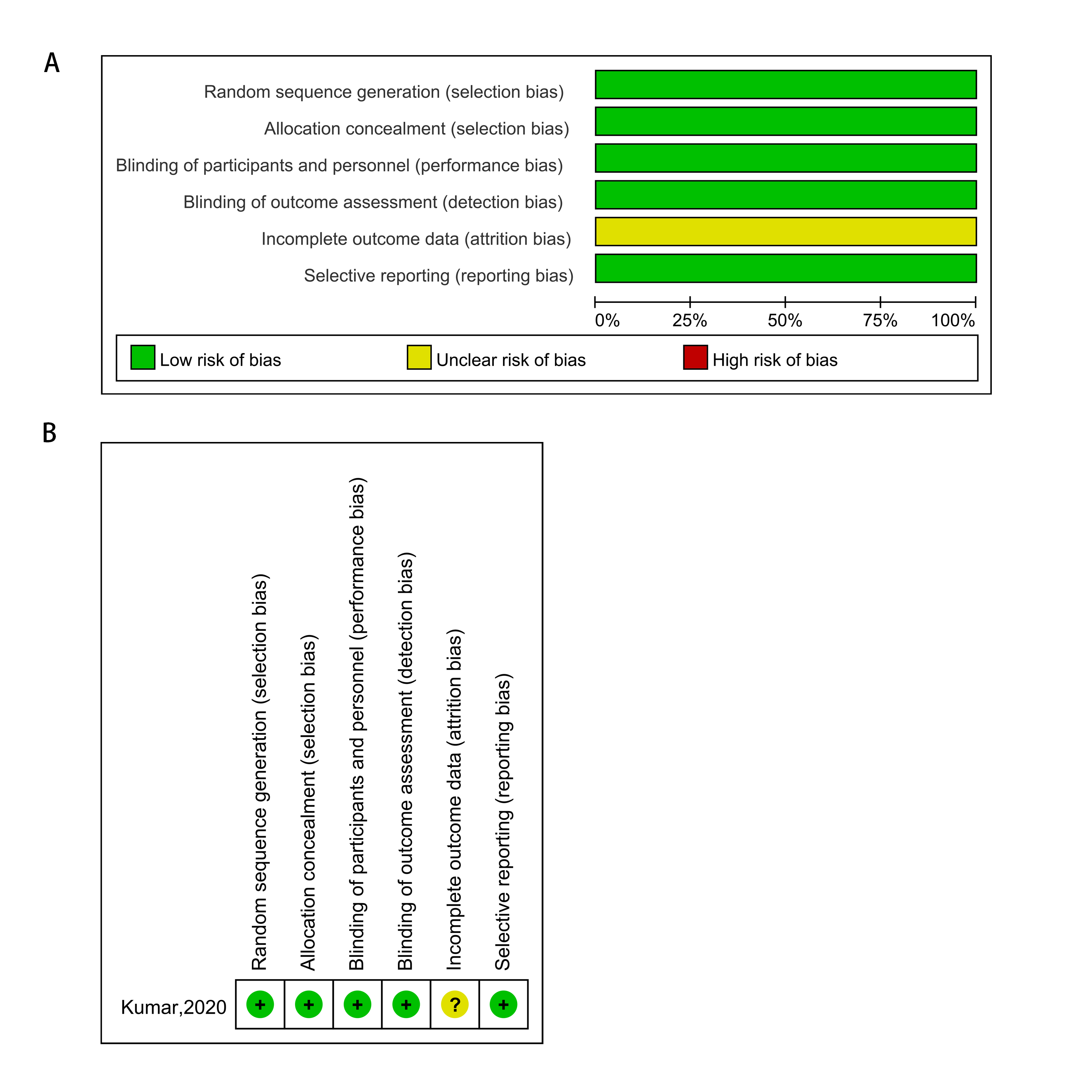

Supplement: Supplemental Material [file IANN_A_2186480_SM7769.tif]
